# Comparative Analysis of Domestic and International Test Guidelines for Various Concrete Repair Materials

**DOI:** 10.3390/ma15093267

**Published:** 2022-05-02

**Authors:** Tae-Kyun Kim, Jong-Sup Park

**Affiliations:** Department of Structural Engineering Research, Korea Institute of Civil Engineering and Building Technology, 283, Goyang-daero, Ilsanseo-gu, Goyang-si 10223, Korea; jspark1@kict.re.kr

**Keywords:** concrete repair, crack-surface treatment method, crack-filling method, crack-injection method, structure protection material, structure restoration material, crack-repair material, durability

## Abstract

The number of aged bridges among concrete structures is increasing. Therefore, to increase their lifespans, repair and reinforcement schemes ought to be implemented. This study selected various repair materials according to crack-surface treatment, crack-filling, and crack-injection methods. These repair materials were evaluated using various test methods proposed by the Korean Standards and the American Society for Testing and Materials for structure protection, structure restoration, and crack repair; the results were analyzed and compared. Consequently, the structure restoration material exhibited a similar freezing–thawing trend as that of chloride, while also exhibiting similar flexural, compressive, bond, and splitting-tensile strengths. However, chloride yielded performance differences (up to two-fold) depending on the type of material used for comparison. The crack-repair material yielded similar trends only for bond strength but yielded differences (up to 2–4-fold) in tensile, compressive, and flexural strengths depending on the material used for comparison. These results confirmed that crack-repair materials exhibit significant differences in performance depending on the manufacturer compared with structure protection and structure restoration materials. Therefore, it is expected that repair materials appropriate for usability, durability, and structure safety, while also being environmentally friendly, could be used in future bridge repairs based on these test evaluations.

## 1. Introduction

The number of aged domestic bridges is increasing rapidly, and the need for extending their lifespans by repairs and reinforcements is emerging. Moreover, significant national budgets will be required for maintaining these bridges in the future [1]. Although, research has been conducted on the developing state and performance evaluation methods for aged bridges and developing elemental techniques for repair and reinforcement methods [2] have been researched [2], the accuracy and effectiveness of the developed techniques and materials have not been verified [2]. Furthermore, highly accurate and effective performance evaluations, repairs, and reinforcements must be performed based on the actual verification of bridges considering the long service lives of rapidly increasing old bridges [2]. However, the available literature still lacks effective verifications. Essentially, repair and reinforcement methods for structures should be proposed considering the durability, waterproofness, safety, homogeneity, and aesthetics of the structure, based on precise safety diagnosis results. In particular, the cause of damage, the scope, and scale of repair, environmental conditions, safety, construction period, and economic efficiency should be considered to achieve the intended objectives [3,4]. Repair and reinforcement objectives vary according to the importance, deterioration, and damage of the deteriorated structure. Nevertheless, the primary objective remains to prevent the deterioration and progression of damage for maintaining the current performances of various attributes, such as safety, durability, and functionality, in addition to restoring the performance of structures already deteriorated and damaged, or those associated with the potential of being damaged [5,6,7,8]. In particular, repair methods used to restore functions other than strength—such as the durability and waterproofness of cracked structures—are classified based on the crack width associated with crack-surface treatment (structure protection, crack repair), crack-filling (structure restoration, crack repair), and crack-injection methods (crack repair) [9]. Furthermore, reinforcement methods used to restore the strength reduction of structural damage owing to cracks include steel-plate reinforcement, continuous-fiber-reinforcement, and external pre-stressing methods. In particular, FRP (Fiber Reinforced Polymer) has been recently used as an alternative material for reinforcement. FRP exhibits high strength, light weight, corrosion resistance, and high durability. The repair and reinforcement of concrete structures is performed using a wide range of materials, such as epoxy for reinforcement, impregnated polymer cement, and waterproofing material for repair [9]. The exposure of these materials to various environmental factors after construction leads to various problems. Therefore, this study aims at analyzing environment, strength, and chemical properties of repair materials, which have not been sufficiently investigated in previous research works.

Kang et al. [10] conducted performance evaluations using crack-injection materials based on inert crack bending specimens using artificial notches. Bae et al. [11] evaluated concrete performance by changing the properties of materials, such as concentration and viscosity, to improve the performance of surface impregnate. Kwon et al. [12] performed impregnate repair on specimens without cracks and evaluated their durability. Christodoulou et al. [13] evaluated the long-term performance of reinforced concrete structures based on surface impregnate silane coatings. Dai et al. [14] evaluated the chloride resistance performance of reinforced concrete according to cracks by using a water-repellent surface impregnate. Previous studies mainly developed repair materials by conducting single experiments on single-parameter materials. Therefore, an in-depth performance analysis for materials properties is needed.

In this study, we perform tests on various materials, including one type of structure protection material, two types of structure restoration materials, and two types of crack-repair materials. Specifically, accelerated carbonation, resistance to chloride ion penetration, and freezing–thawing tests are performed for structure protection materials. Flexural, compressive, bond, resistance to chloride ion penetration, and splitting-tensile strength tests are performed for material structure restoration. Furthermore, bond strength, tensile strength, elongation, compressive strength, and flexural strength tests are performed for crack-repair materials. Therefore, long- and short-term performance tests were carried out, and analyses of concrete structure repair materials were conducted to address the issues reported in previous studies.

In practice, damaged structures are actually repaired at construction sites using materials and test methods provided by manufacturers. However, provided materials need to be reviewed to evaluate whether the performance of the repair materials is sufficient for the actual case. Moreover, domestic test standards are followed for evaluating long- and short-term performances. If comparative analysis with overseas tests is possible, the performance of aged concrete and bridge repair materials is measured by adhering to domestic and overseas test standards. These tests are performed using materials from various manufacturers according to structure protection, structure restoration, and crack repair using Korean Standards (KS) and American Society for Testing and Materials (ASTM) standards. The number of test specimens for each type of experiment shall be 3 to 5, respectively. Furthermore, the performance of materials after repair and the feasibility of quality are analyzed. It is expected that materials suitable for the environmental characteristics, usability, durability, and safety of the structures will be used for actual bridge repairs in the future. In particular, this study focuses on identifying the materials repair capability adopted in various environmental conditions instead of their general-purpose detailed properties and chemistry.

## 2. Materials and Methods

### 2.1. Structure Protection Materials

Table 1 summarizes the advantages and disadvantages of structure protection materials. Although these vary according to the purpose of repair and the environment of structures, the materials used in the structure protection method should have an adequate performance to prevent or control the penetration of foreign substances, such as CO_2_, moisture, and chloride ions. Moreover, they are required to adequately resist the adverse effects from the inside and outside of the structure and have excellent workability during construction. The permeable absorption impregnate (inhibitor) is applied and impregnated onto the concrete surface to form an absorption prevention layer. Its main role is to suppress the infiltration of water, CO_2_, and chloride ions from the outside. In particular, silicon-based permeable absorption agents are frequently used. A permeable solidifying material is applied to the concrete surface, solidifying the part with low durability and integrating it with the sound part. Furthermore, when an inorganic, permeable waterproofing material is applied to the concrete surface layer, silicate ions from the fine silicate powder (water-soluble silica) in the material penetrate the concrete to form insoluble crystals by reacting with calcium ions in the pores inside the concrete, thus forming a dense waterproofing surface layer [9].

### 2.2. Structure Restoration Material

Table 2 lists the types of structure restoration materials (repair mortar). Typically, the repair aims at restoring the functions of damaged concrete structures, such as cracking, peeling, exfoliation, or rebar corrosion due to various performance degradation factors. In other words, it refers to the act of restoring the properties of concrete by maintaining or improving its unique functionality imparted at the beginning of service to prevent problems from occurring during the service periods of structures. Numerous repair techniques are being applied based on the used material or development principle. Among various processing methods, the cross-sectional restoration (repair) method is used for the structural restoration of concrete structures [9].

### 2.3. Crack-Repair Materials

Table 3 lists the types of crack-repair materials (crack-injection materials). Resin-based and cement-based materials are mainly used in injection methods, with epoxy resin-based materials being used extensively. The epoxy resin injection method is excellent in terms of injection property, curing speed, and adhesiveness. However, wet surfaces constitute a limitation for the application of this method because the required adhesiveness is not secured. In addition, epoxy resin exhibits differences in thermal expansion coefficient from concrete.

Meanwhile, cement-based injection materials have similar properties, including the elastic modulus and thermal expansion coefficient to existing materials, with several advantages: excellent adhesiveness on wet surfaces, local rebar corrosion inhibition effects, and relatively lower costs. However, they also have disadvantages associated with the difficulty of injecting cement in fine cracks (around 0.05 mm) and typically have long curing times and low adhesiveness [9].

As described above, resin- and cement-based injection materials have their own advantages and disadvantages. Hence, it is advisable to choose a repair material for reinforced concrete structures based on considerations of the properties of materials after clearly setting the purpose of use.

## 3. Performance Tests for Structure Protection Materials

### 3.1. Performance Analysis of Structure Protection Material (KS, ASTM)

Structure protection materials protect the members and improve the durability of concrete structures by modifying the structure of the concrete surface layer and imparting special functions through a surface impregnate layer. Surface impregnates are classified into three types: Silane-based, silicate-based, and others. The surface impregnation method could be applied for the following purposes: carbonation, chloride, frost damage prevention, alkali aggregate reaction, aesthetics, landscape, and waterproofing, depending on the required performance. Thus, a surface impregnate that satisfies each performance requirement should be used. The performance analysis test for structure protection materials is performed using a liquid penetrating impregnate according to the test standards and methods listed in Table 4.

### 3.2. Structure Protection Material Tests and Results

#### 3.2.1. Accelerated Carbonation

**Figure 1:**
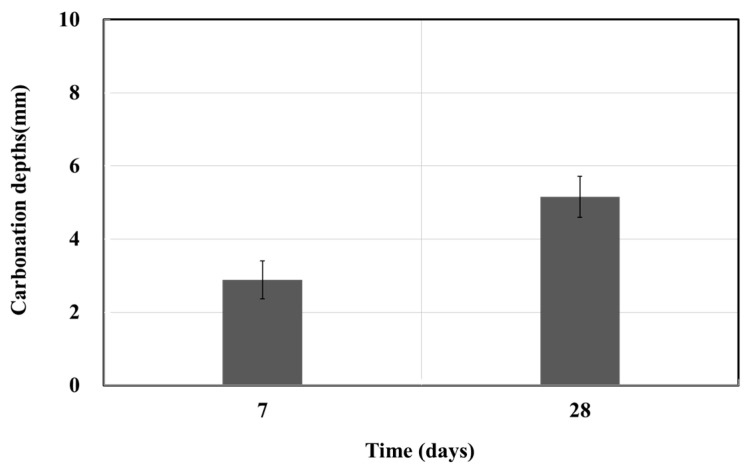
Accelerated carbonation depth.

The accelerated carbonation test was performed in accordance with KS F 2584 (Standard test method for accelerated carbonation of concrete) and KS F 2596 (Method for measuring carbonation depth of concrete) [15,16]. The specimen was placed inside a thermo–hygrostat chamber at a temperature of 20 °C, relative humidity (RH) of 60%, and CO_2_ concentration of 5%. The depth of color changes was then measured after 1% phenolphthalein was sprayed for a curing period which ranged from 1 to 4 weeks. Figure 1 shows the accelerated carbonation test results. At weeks 1 and 4, the carbonation depths were approximately 3.2 mm and 4.64 mm, respectively. By week 4, it penetrated a depth of 1.3 mm, confirming the effect of the penetrating impregnate over time. In the accelerated carbonation test, a product of the construction new technology manufacturer B was used, with the penetration depth exceeding the specified value.

After 4 weeks, the penetration of carbon dioxide exceeded the quality standard of 1.0 mm because of the quality errors of the products produced by the manufacturer. There is no problem when mixing the impregnate by weight, but there is a concern for poor mixing when mixing is conducted based on volume; thus, special care should be exercised. Accordingly, quality inspection must be performed because the accelerated carbonation measured in this test yields significant differences from the specified values.

#### 3.2.2. Chloride of Structure Protection Material

The resistance to chloride ion penetration test was performed in accordance with KS F 2711 (Standard test method for resistance of concrete to chloride ion penetration by electrical conductance) and ASTM C 1202 (Standard test method for electrical indication of concrete ability to resist chloride ion penetration) [17,18]. Using an aqueous 0.3 M NaOH solution at a temperature of 20 °C with RH of 60% at the anode and a 3% NaCl aqueous solution at the cathode, the current applied to the resistance of 0.2 Ω and the temperature inside the diffusion cell with an applied voltage of 60 V was measured for 6 h. The penetration depth of chloride ions was measured using Vernier calipers in the area discolored by splitting the specimen and spraying silver nitrate solution after the test.

**Figure 2:**
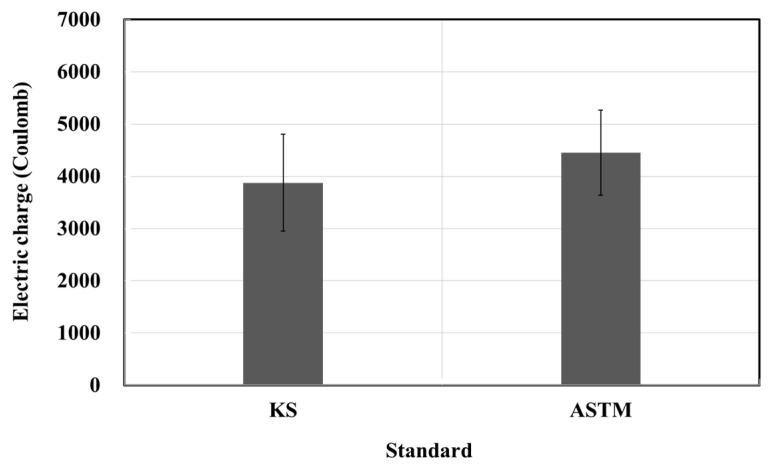
Resistance to chloride ion penetration electric charge test results based on the KS and ASTM standards.

**Figure 3:**
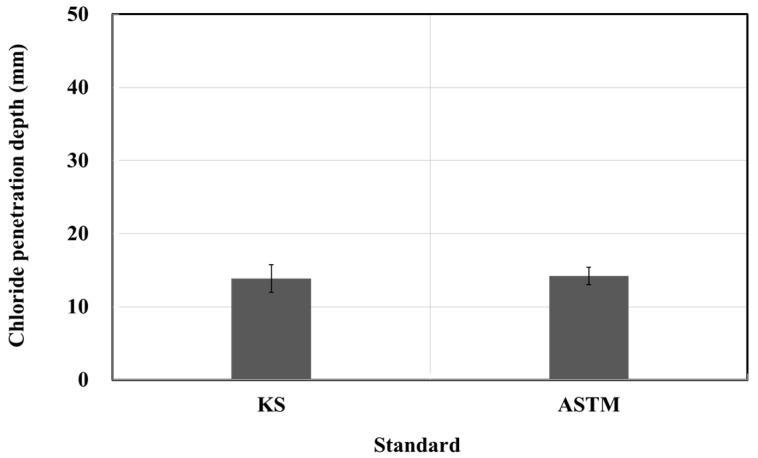
Chloride penetration depth test results based on the KS and ASTM standards.

Figure 2 and Figure 3 show the results of the resistance to chloride ion penetration test. The electric charge was 3878 C for the KS standard and 4453 C for the ASTM standard [17,18]. The penetration depth was 13.88 mm for the KS standard and 14.21 mm for the ASTM standard. Thus, the electric charge and penetration depth of the ASTM are 1.14 and 1.02 times higher than those of the KS, respectively. Moreover, the electric charge reference value of the resistance to chloride ion penetration suggested by the manufacturer was 1000 C, but the test results were 3.8 and 4.4 times larger than the specified values, attributed to the quality errors of the products produced by the manufacturer. There is no problem when mixing the impregnate based on weight, but there is a concern for poor mixing when mixing based on volume, and special care should be taken. In particular, considering that the resistance to chloride ion penetration is affected significantly by the environmental conditions, which in turn affects durability, these quality parameters must be considered in detail.

#### 3.2.3. Freezing-Thawing Test

The resistance to the freeze-thaw test was performed in accordance with KS F 2456 (Standard test method for resistance of concrete to rapid freezing and thawing) and ASTM C 666 (Standard test method for resistance of concrete to rapid freezing and thawing)—test methods that freeze in air and thaw in water [19,20]. Freezing and thawing in the temperature range of −18~4 °C for 4 h was set to occur in one cycle. The tests were conducted for 0, 100, 200, and 300 cycles, and the relative dynamic elastic modulus and durability factor of the concrete were measured by resonance vibration. Equation (1) is the expression for the relative dynamic elastic modulus and Equation (2) is the expression for the durability factor,

(1) $$P_{c}=\left(\frac{n_{c}^{2}}{n_{0}^{2}}\right)\times 100,$$

where $P_{c}$ is the relative dynamic elastic modulus (%) after *C* freeze-thaw cycles, $n_{c}$ is the first resonance frequency (Hz) of the strain vibrations at the 0th freezing-hawing cycle, and $n_{o}$ is the first resonance vibrations (Hz) of the strain vibration after *C* freeze-thaw cycles.

(2) DF=PN/M,

where *DF* is the durability factor of the test specimen, *P* is the relative dynamic elastic modulus (%) in *N* cycles, *N* is the number of cycles counted either when the relative dynamic elastic modulus reaches 60%, or at the moment the freezing-thawing test finishes. M is the number of cycles when the freezing-thawing finishes.

**Figure 4:**
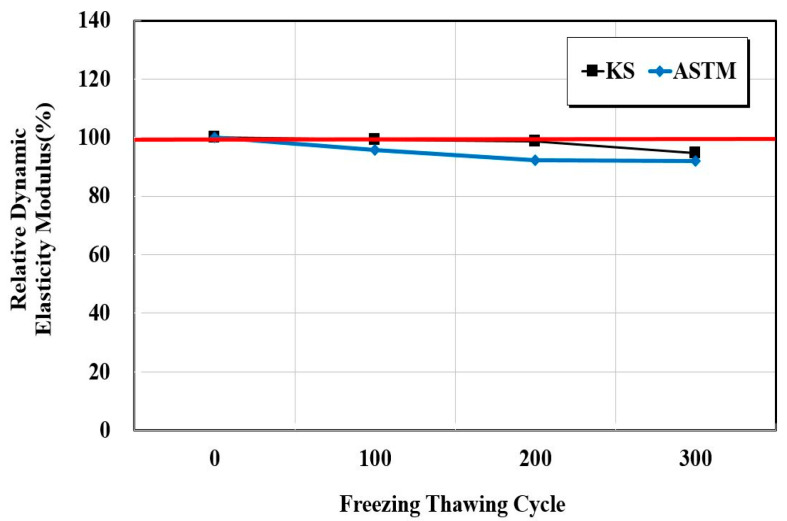
Relative dynamic elastic modulus test results as a function of the freezing–thawing cycle.

**Figure 5:**
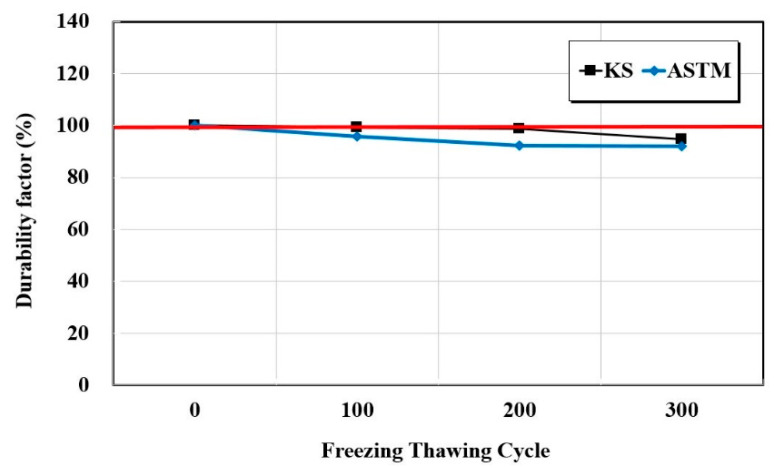
Durability factor test results as a function of the freezing–thawing cycle.

**Figure 6:**
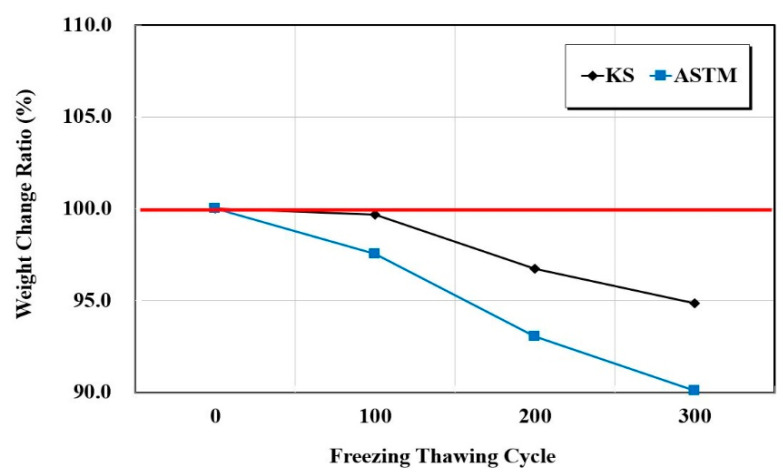
Weight change ratio test results as a function of the freezing–thawing cycle.

**Figure 7:**
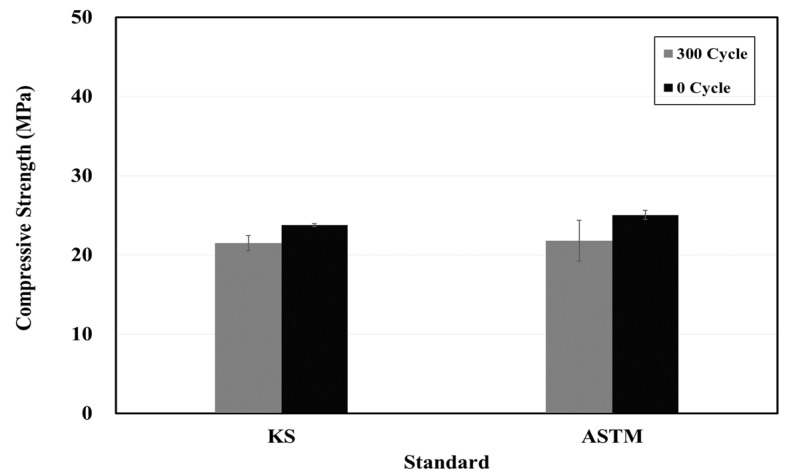
Compressive strength test results based on the KS and ASTM standards at the 0th and 300th freezing–thawing cycles.

Figure 4, Figure 5 and Figure 6 show the resistance responses to the freezing-thawing tests. Furthermore, Figure 7 shows the compressive strength after freezing–thawing. The relative dynamic elastic modulus was higher than 90% in both KS and ASTM standards, and the durability factor showed similar trends. Moreover, a weight change within 10% occurred at the weight change ratio. The surface impregnate showed an excellent freezing–thawing resistance performance. Between the KS and the ASTM, the values of the relative dynamic elastic modulus and durability factor changes of the ASTM were approximately 0.97 times smaller than those of the KS, and the weight change ratio was 0.95 times smaller. The test result of the KS compressive strength was approximately 28.8 before the freezing-thawing test and 21.5 after 300 cycles. Thus, the compressive strength decreased by approximately 10%. The test result of the ASTM compressive strength was also approximately 25.0 before the freezing-thawing test and 21.8 after 399 cycles, thus yielding a reduction of compressive strength of approximately 13%. Consequently, both KS and ASTM yielded excellent freezing-thawing test results. Furthermore, the resistance to freezing and thawing (relative dynamic elastic modulus) was higher than 80% of the general standard value of concrete quality, thus indicating good quality.

## 4. Performance Test of Structure Restoration Materials

### 4.1. Structure Restoration Material Performance Analysis (KS, ASTM)

Structure restoration materials were used to fill a defect area after removing an unsound area of concrete to eliminate problems of adhesion to concrete and the intrusion of deterioration factors. Polymer-cement mortar was mainly used with spray and prepack concrete methods applied in case of large defect areas. Furthermore, for cross-sectional restoration after exposure of the rebar, such as the rebar antirust treatment, polymer cement mortar, and concrete, which have material properties close to cement and concrete, are often used considering the corrosion of rebar. Lastly, when repairing structures with a large repair cross-section, a structure restoration material that has similar mechanical properties as the concrete of the repair part needs to be selected. The performance analysis test used for structure restoration materials was performed in accordance with the test standards and methods based on two types of polymer cement repair mortars, as shown in Table 5.

### 4.2. Structure Restoration Material Test and Results

#### 4.2.1. Flexural Strength of Structure Restoration Materials

**Figure 8:**
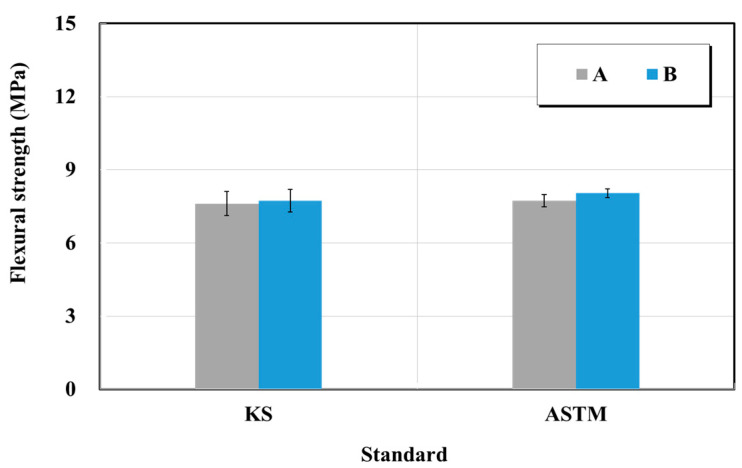
Flexural strength test results of structure restoration materials (repair mortar) obtained based on the KS and ASTM standards.

Structure restoration material products A and B from a manufacturer in South Korea were used in this test. The flexural strength test was conducted in accordance with KS F 4042 (Polymer modified cement mortar for maintenance in concrete structure flexural strength test) and ASTM C 348 (Standard test method for flexural strength of mortars) [21,22]. The flexural strength specimen was produced by slowly pouring the fabricated sample into a 40 × 40 × 160 mm^3^ mold and curing it at a temperature of 20 ± 2 °C with RH of 65 ± 10%. The sample was demolded 24 h after pouring into the mold, and the flexural strength was measured after 28 d. Figure 8 show the test results. The two products yielded the flexural strengths of 7.6 and 7.7 MPa (product A), and 7.7 and 8.0 MPa (product B) in the cases of the KS and ASTM standards, respectively. Thus, they satisfied the specifications suggested by the manufacturer by achieving the standard value of 6.0 MPa or higher specified by the KS quality standard. Compliance with the ASTM C 348 standard also yielded a similar result trend as that of the KS. Both manufacturers A and B showed highly similar changes of flexural strengths equal to 1.01 and 1.03 times in KS and ASTM.

#### 4.2.2. Compressive Strength of Structure Restoration Materials

**Figure 9:**
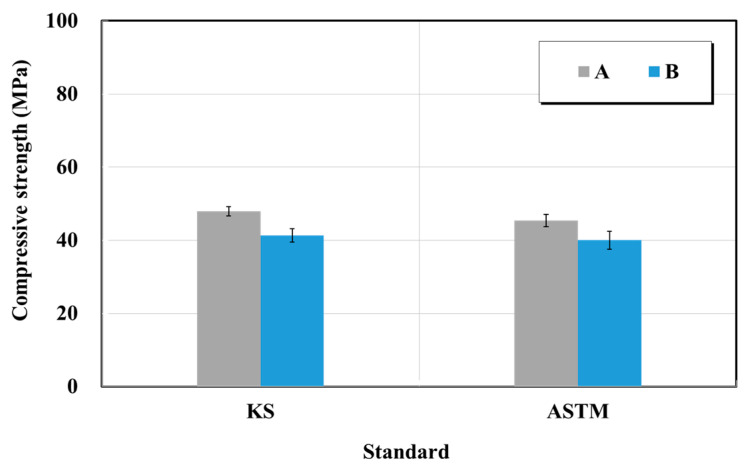
Compressive strength test results of structure restoration materials (repair mortar) obtained based on the KS and ASTM standards.

The compressive strength test was performed in accordance with KS F 4042 (Polymer modified cement mortar for maintenance in concrete structure—compressive strength test) and ASTM C39/C39M (Standard test method for compressive strength of cylindrical concrete specimens) [21,23]. The compressive strength specimen is the specimen after the flexural strength test, and the compressive strength is measured using a 40 × 40 × 40 mm^3^ pressure plate. Figure 9 shows the compressive strength test results. Based on the tests, the two products yielded the compressive strengths of 47.9 and 45.4 MPa (product A), and 41.3 and 40 MPa (product B) corresponding to the KS and ASTM standards, respectively. Furthermore, they satisfied the specification value suggested by the manufacturer and the standard value by achieving the KS quality standard of ≥ 20 MPa. The trend when using the ASTM C 39 standard was similar to that obtained in the KS case. The changes of flexural strength in the KS and ASTM cases were also highly similar at 0.86 and 0.88 times. Thus, the ASTM standard can also be followed safely as the compressive strength test outcome based on this standard was similar to that of the KS standard. Furthermore, the KS F 4042 standard was the same as the KS L ISO 679 standard. Thus, the test method used for the quality test items is valid as an international standard.

#### 4.2.3. Bond Strength of Structure Restoration Materials

The bond strength test was conducted in accordance with KS F 4042 (Polymer modified cement mortar for maintenance in concrete structure—bond strength test) and ASTM C1404/C1404M (Standard test method for bond strength of adhesive systems used with concrete as measured by direct tension) [21,24]. For the bond strength test, a metal or synthetic resin mold with an inside dimension of 40 × 40 × 10 mm^3^ was inserted after polishing the test base, and the sample was molded by filling it to the same height as the mold. It was then cured at 20 ± 2 °C at RH of 65 ± 10%. At 24 h after molding, it has been demolded and cured again for 28 d to obtain the bond strength specimen. Furthermore, as it is challenging to fabricate bond strength specimens according to the ASTM standard, an alternative test was performed in accordance with the KS test standard using a ∅50 attachment. The method of testing a pull-out load by attaching a circular attachment to the base material is considered valid. A specimen that had been cured, as specified, was placed horizontally in the curing room, and an adhesive was applied on the sample surface. An upper jig was placed for the tensile tests (steel) and was attached by rubbing it lightly. The maximum tensile load was then obtained by applying a tensile force perpendicular to the sample plane using a lower jig for the tensile test (steel) and a steel-base plate.

**Figure 10:**
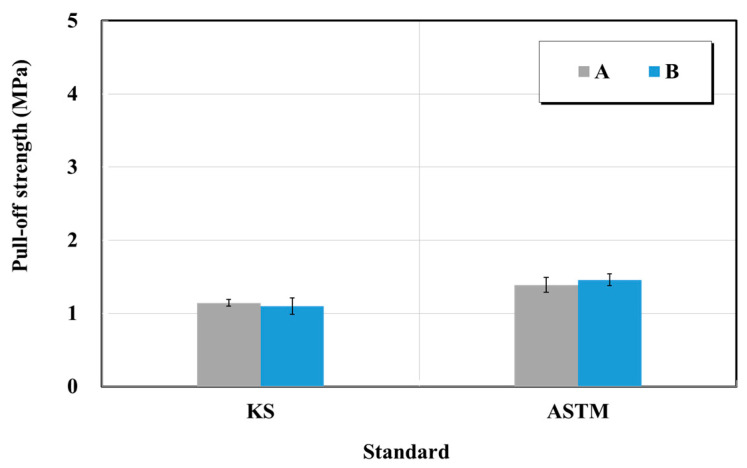
Bond strength test results of structure restoration material (repair mortar) obtained based on the KS and ASTM standards.

Figure 10 shows the bond strength test results, based on which the bond strength was 1.1 MPa in the KS standard case, and the bond strengths of the two products in the ASTM standard case were 1.4 and 1.5 MPa, respectively. Hence, they satisfied the specification suggested by the manufacturer and the KS quality standard of 1.0 MPa or higher. In addition, products A and B exhibited highly similar flexural strength changes corresponding to the KS and ASTM standards.

#### 4.2.4. Splitting-Tensile Strength

**Figure 11:**
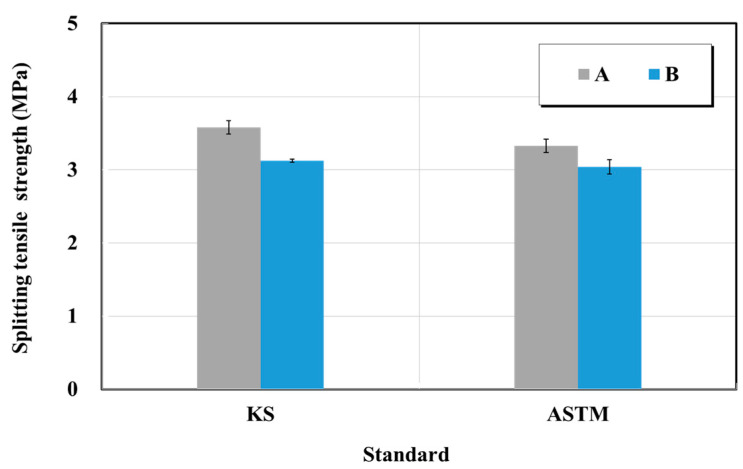
Splitting tensile strength test results of structure restoration material (repair mortar) obtained based on the KS and ASTM standards.

The splitting tensile strength test was performed in accordance with KS F 2423 (Standard test method for splitting tensile strength of concrete) and ASTM C 496 (standard test method for splitting tensile strength of cylindrical concrete specimens) [25,26]. Figure 11 shows the results of the splitting tensile strength test. The splitting tensile strengths were 3.6 and 3.3 MPa (product A) and 3.1 and 3.0 MPa (product B) corresponding to the KS and ASTM standards, respectively. Furthermore, the changes of the two products in the KS and ASTM standards were similar at 0.91 and 0.96 times, respectively. There is no specification suggested by the manufacturer and no KS quality standard for the splitting-tensile strength. In general, the splitting-tensile strength of ordinary concrete considers values equal to those in the range of 1/10–1/13 of the compressive strength. If the result of the splitting-tensile strength test is examined based on this predicted result, the compressive strength test result was in the range of 40–45 MPa, and the splitting-tensile strength was 3.0 MPa or higher, suggesting good quality specimens.

#### 4.2.5. Carbonation

**Figure 12:**
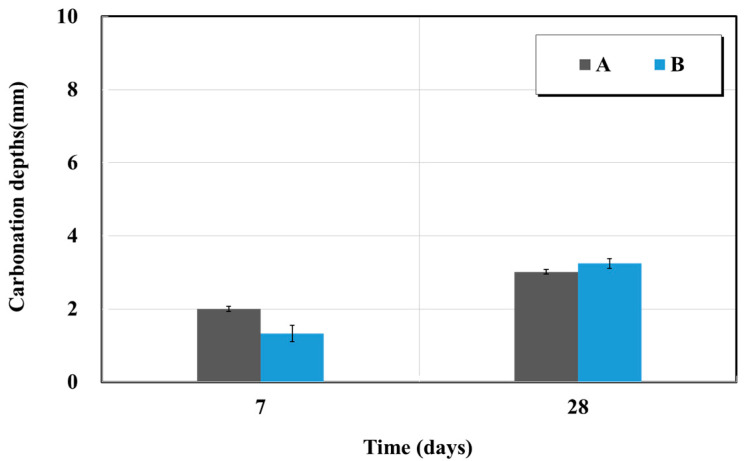
Accelerated carbonation depth test results as a function of time.

The accelerated carbonation test was performed in accordance with KS F 2584 (Standard test method for the accelerated carbonation of concrete) and KS F 2596 (Method for measuring carbonation depth of concrete) [15,16]. A 1% phenolphthalein spray test was performed after the specimen was inserted in the CO_2_ thermo–hygrostat at the temperature of 20 °C, RH of 60%, and CO_2_ concentration of 5%. The carbonation depth was measured by the depth of color change at weeks 1 and 4. Figure 12 shows the results of the accelerated carbonation test. For product A, the carbonation depth was approximately 2.0 mm at week 1 and approximately 3.01 mm at week 4; the corresponding values for product B were approximately 1.33 mm and 3.25 mm. Thus, products A and B yielded values equal to 1.5 and 2.5 times the penetration depth over time.

The sectional restoration materials used in this accelerated carbonation test were the patented product A, and the constructed product B. They penetrated more than the specification value suggested by the manufacturer. After 28 d (week 4), CO_2_ penetration exceeded the KS 4042 quality standard value of 2.0 mm. As time progressed, product A yielded a high performance as a function of the penetration depth ratio [21].

#### 4.2.6. Chloride of Structure Restoration Materials

The resistance to chloride ion penetration test was performed in accordance with KS F 2711 (Standard test method for resistance of concrete to chloride ion penetration by electrical conductance) and ASTM C 1202 (Standard test method for electrical indication of concrete ability to resist chloride ion penetration) [17,18]. Using a 0.3 M NaOH aqueous solution at a temperature of 20 °C and RH of 60% at the anode and a 3% NaCl aqueous solution at the cathode, the current applied to a resistance of 0.2 Ω and the temperature inside the diffusion cell was measured for 6 h. In addition, the penetration depth of the chloride ion was measured using a Vernier caliper for the discolored part when the specimen was split and after a 0.1 N AgNO_3_ solution was sprayed after the test finished.

**Figure 13:**
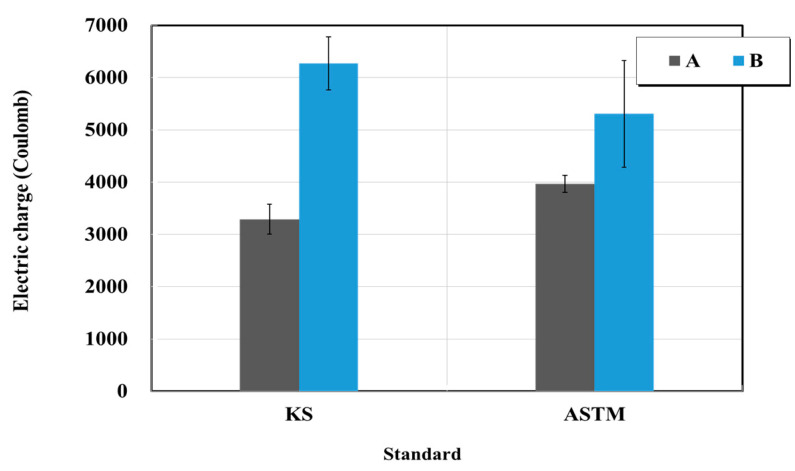
Results of electric charge test for resistance to chloride ion penetration obtained based on the KS and ASTM standards.

**Figure 14:**
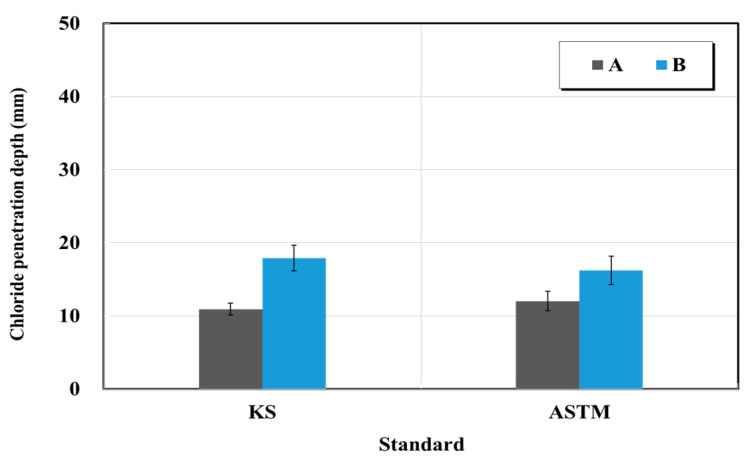
Chloride penetration depth test results obtained based on the KS and ASTM standards.

Figure 13 and Figure 14 show the results of the resistance to chloride ion penetration test for structure restoration materials (sectional restoration materials). The electric charges of product A in the cases of the KS and ASTM standards were 3290 and 3968 C, respectively. The electric charge of the product of B corresponding to the KS and ASTM standards was 6272 and 5304 C, respectively. Furthermore, the chloride penetration depths of product A in the cases of the KS and ASTM standards were 10.95 and 12.05 mm, respectively. The chloride penetration depths of product B in the KS and ASTM standards were 17.93 and 16.25 mm, respectively. In addition, when the changes of KS and ASTM by the manufacturer were examined, the changes of the electric charge for A and B were 1.2 times higher and 0.8 times lower, respectively. In the case of the chloride penetration, the changes of A and B were 1.1 times higher and 0.9 times lower, respectively. The changes of the standards KS and ASTM associated with different manufacturers sometimes appear to be opposite. Therefore, the resistance to chloride-ion penetration measured in this test significantly differs from the standard values, thus requiring quality inspection.

## 5. Performance Test of Crack Repair Materials

### 5.1. Analysis of Crack-Repair Material Performance (KS, ASTM)

The crack repair techniques are applied when cracks occur in existing or new concrete structures, and are classified into crack-surface-treatment, crack-injection, and crack-filling methods. The crack-surface-treatment method is typically used for fine cracks smaller than 0.2 mm. It improves waterproofness and durability by forming a coating film, which covers the cracked part. The crack-injection methods are typically used when the crack is larger than 0.2 mm, and improve waterproofness and durability by injecting low-viscosity resin-based or cement-based materials inside the cracks. These methods are classified into low- and high-pressure types depending on the used pressure. The crack-injection method consists of crack cleaning treatment, pipe setting for injection, crack-surface sealing, injection, pipe removal, and seal removal. Organic and polymer cement materials are typically used. The crack-filling method is appropriate for repairing relatively large crack widths (≥0.5 mm). It is a method involved with the cutting mortar finish or concrete along the crack and with the filling of the damaged part with a repair material. The filling materials used for this method are generally organic, polymer cement, and cement materials. The crack repair material performance analysis test is performed by test standards and methods based on two types of materials as shown in Table 6.

### 5.2. Crack Repair Material Test and Result

#### 5.2.1. Tensile Strength and Elongation

The tensile strength test (elongation at tensile fracture) was performed in accordance with KS F 4923 (Epoxy adhesives for repairing in concrete structure) and KS M ISO 527 (Tensile strength and elongation at tensile failure, Plastics–Determination of tensile properties, general principles (Part 1), test conditions for molding and compressive plastics) and ASTM D 638 (Standard test method for tensile properties of plastics) [27,29,31].

**Figure 15:**
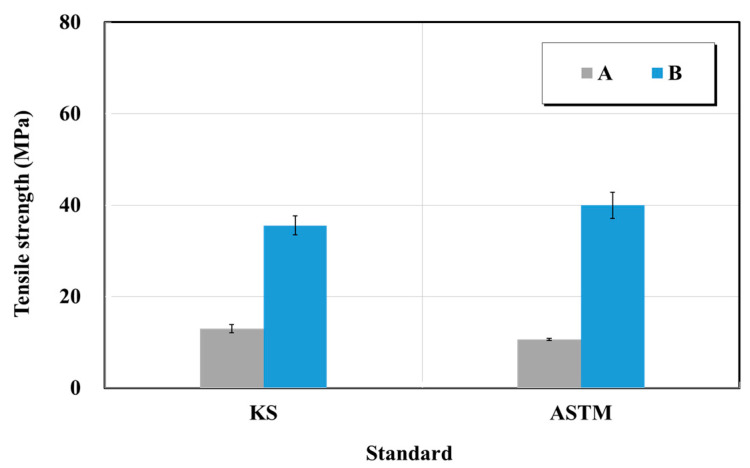
Tensile strength test results of crack-injection materials (epoxy resin) based on the KS and ASTM standards.

**Figure 16:**
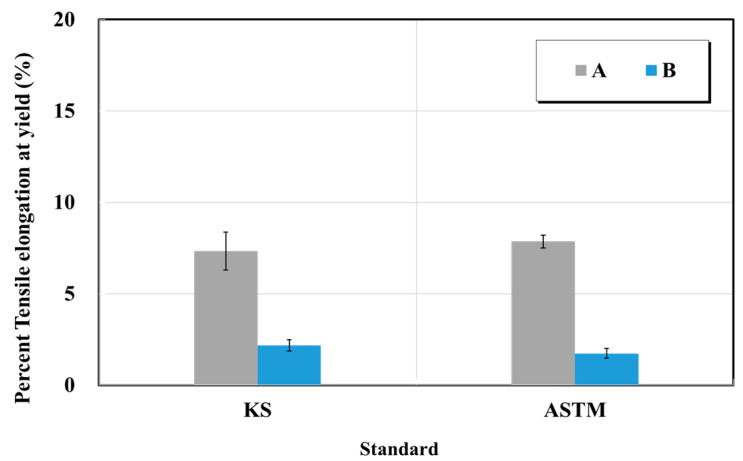
Elongation at tensile fracture test results of crack-injection materials (epoxy resin) based on the KS and ASTM standards.

Crack-injection materials products A and B were used as specimens. Each of the epoxy-impregnated resin materials and curing agent were mixed and molded immediately after mixing, and tensile tests were performed five times each at 1 mm/min. Figure 15 and Figure 16 show the results of the tensile strength and elongation tests for the epoxy-impregnated resins from manufacturers A and B. The rated tensile strength of the epoxy impregnated resin in the catalog of manufacturer A is 43 MPa. The tensile strength of the epoxy impregnated resin on the catalog of manufacturer B was 15 MPa or higher. The quality standard in previous studies was 15 MPa in the case of the epoxy for crack repair (rigid type). As a result of this test, the tensile strength of the epoxy impregnated resin A was in the range of 11–13 MPa, and did not satisfy the quality standard. The tensile strength of the epoxy impregnated resin B was in the range of 36–40 MPa. Product B exceeded both the quality standard and the value of the manufacturer.

The quality standard of the elongation at tensile fracture was 10% lower, and both products satisfied it. Product A yielded a lower tensile strength than the quality standard. This is attributed to the fact that when a tensile specimen was fabricated, the bubbles generated when the main material and hardener were mixed affected the strength of the tensile strength. Removing the bubbles is a key step toward the reduction of the deviations of the test. Furthermore, it was confirmed that there were no deviations in test results between the KS and ASTM standards. However, the test results yielded a 34-fold difference according to the specifications of each manufacturer.

#### 5.2.2. Flexural Strength of Crack Repair Materials

**Figure 17:**
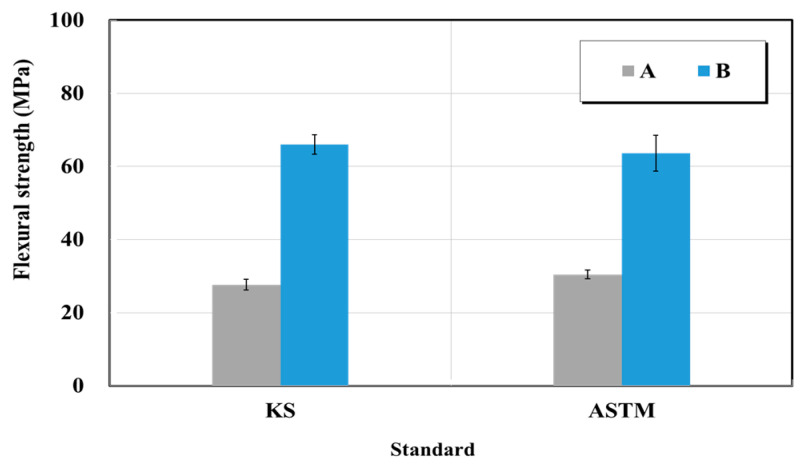
Flexural strength test result of crack-injection materials (epoxy resin) based on the KS and ASTM standards.

The flexural strength test was performed in accordance with KS M 3015 (Testing methods for thermosetting plastics) and ASTM D 790 (Standard test methods for flexural properties of unreinforced and reinforced plastics and electrical insulating materials) [30,34]. Figure 17 shows the flexural strength test result for the epoxy-impregnated resins from manufacturers A and B. Based on the test, the epoxy-impregnated resin flexural strengths of product A were 28 and 30 MPa when the KS and ASTM standards were followed, respectively. The epoxy-impregnated resin flexural strengths of product B were 66 and 64 MPa based on the KS and ASTM standards, respectively. This trend was similar to the flexural strength quality standards for epoxy resin for concrete attachment. Similar to the tensile strength, the removal of bubbles is the key step for the reduction of the test deviations because the bubbles generated when the main material and hardener are mixed when fabricating the epoxy resin flexural test specimens, affect the strengths of the flexural test specimens.

Moreover, both specimens from manufacturers A and B finally yielded a failure bending mode instead of fracture. Regarding the test method, no deviations were found in the test outcomes obtained based on the KS and ASTM standards. However, the deviations between manufacturers and tensile strengths were considerable.

#### 5.2.3. Compressive Strength of Crack Repair Materials

**Figure 18:**
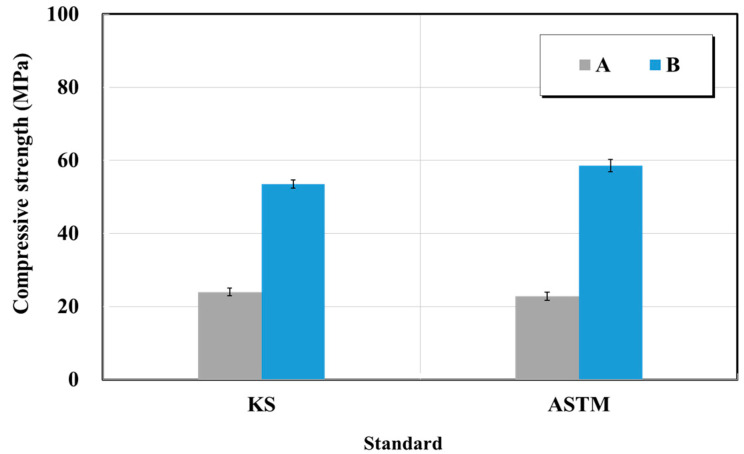
Compressive strength test results of crack-injection materials (epoxy resin) based on the KS and ASTM standards.

The compressive strength test was performed in accordance with KS F 4923 (Epoxy adhesives for repairing in concrete structure), KS M ISO 844 (Compressive strength of rigid epoxy resin rigid cellular plastics—Determination of compression properties), and ASTM D 695 (Standard test method for compressive properties of rigid plastics) [27,32,34]. Figure 18 shows the compressive strength test results of the crack repair materials (epoxy resin) of products A and B. The compressive strength of the epoxy impregnated resin on the catalog of A was 118 MPa, and the compressive strength of the epoxy impregnated resin listed in the catalog of B was ≥50 MPa. As a result of this test, the epoxy impregnated resin compressive strengths of product A were 24 and 23 MPa, respectively, which did not satisfy the quality standard. The epoxy-impregnated resin compressive strengths of product B were 54 and 59 MPa, respectively. These values exceeded both the quality standards and the specifications of the manufacturer. The cause of the lower compressive strength for product A compared with the quality standard is similar to that of the tensile and flexural strengths. Furthermore, the compressive strength requires flat top and bottom specimen parts. However, it has been shown that the epoxy-impregnated resin is deformed owing to shrinkage that occurs during its curing and grinding to match the smoothness that affected the accuracy of the test. Moreover, there were no deviations associated with the test results between the test methods of KS and ASTM standards.

#### 5.2.4. Bond Strength of Crack Repair Materials

**Figure 19:**
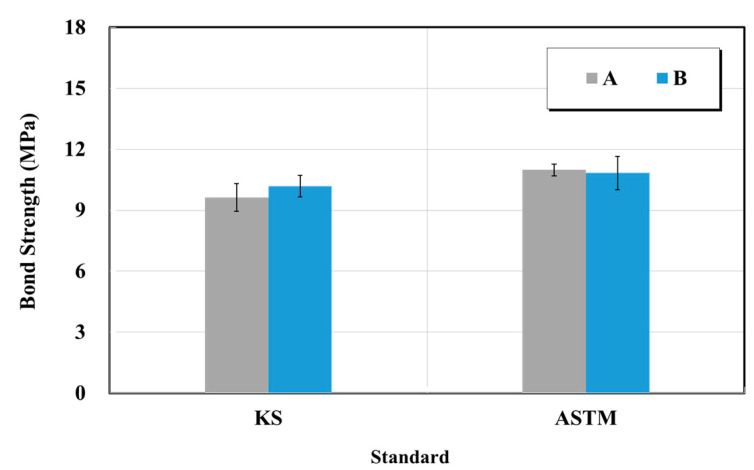
Bond strength test results of crack-injection materials (epoxy resin) based on the KS and ASTM standards.

The bond strength test was performed in accordance with KS F 4923 (Epoxy adhesives for repairing in concrete structure) and ASTM C 882 (Standard test method for bond strength of epoxy-resin systems used with concrete by slant shear) [27,28]. Figure 19 shows the bond strength test results of the epoxy-impregnated resins from manufacturers A and B. As a result of the test, the epoxy-impregnated resin bond strengths for A and B according to the KS and ASTM standards were 9.6 and 11.0 MPa, and 10.2 and 11.0 MPa, respectively. It can be observed that similar results were obtained for bond strength in all conditions according to manufacturer and test standards.

## 6. Conclusions

In this study, domestic and international standard tests were performed using various repair materials, including structure protection, structure restoration, and crack-repair materials according to the repair method. The main conclusions derived from this study are as follows.

1. When the changes of KS and ASTM were compared for the surface impregnate of the structure protection materials, the values of ATM were 1.14 and 1.02 times higher for the chloride penetration resistance electric charge and penetration depth, respectively. When the relative dynamic elastic modulus, durability factor, and weight change ratio during the final 300 cycles of the freezing–thawing process were compared with the 0th cycle, the values of ATM were 0.97, 0.97, and 0.95 times lower, respectively. After the freezing–thawing process, the compressive strength changes were 0.9 and 0.88 times lower in the KS and ASTM cases, respectively. Thus, the decrease in the compressive strength of the ASTM was larger.
2. When the changes of KS and ASTM for structure restoration materials A and B were compared, flexural, compressive, bond, and splitting tensile strengths showed similar trends. However, when carbonation depths at 7 and 28 days were compared, it was 1.5 times for manufacturer A and 2.45 times for manufacturer B. Thus, the carbon dioxide penetration depth increased over time for the material obtained from manufacturer B. The chloride penetration resistance electric charge and penetration depth of manufacturer A increased by approximately 1.18 times, but those of manufacturer B decreased by approximately 0.8 times.
3. When the changes of KS and ASTM for structure restoration materials A and B were compared, the tensile and compressive strengths appeared 0.85 times lower and 1.11 times higher, respectively. The changes in flexural strength were 1.07 times lower and 0.9 times lower, respectively. Thus, the changes in test standards of materials appeared similar, but the difference increased up to 2 to 4 times when materials A and B were compared by using the same test method. The bond strength showed similar trends by material and test method. Thus, the structure restoration materials yielded larger performance differences by manufacturer than the structure protection materials. Therefore, more accurate material tests should be performed for structure restoration materials when they are used for repairs.
4. In this study, three, five, and four test methods of KS and ASTM were comparatively analyzed for one type of structure protection, two types of structure restoration, and two types of crack-repair materials, respectively. In particular, this study identified the repair capability of materials in various environmental conditions such as chloride ion penetration, carbonation, freeze-thaw, and loading, which have been insufficiently researched. However, more repair materials will be used in a follow-up study. In addition, more test methods and the international standards of the International Standardization Organization, American Concrete Institute, and European Norm will be applied for more accurate and precise analyses of materials.

## Figures and Tables

**Table 1:** Advantages and disadvantages of structure protection materials.

Components	Advantages	Disadvantages
Epoxy resin	Good chemical resistance Good water resistance	Low-weather resistance Low variability Poor hardenability at low temperature
Vinyl ester resin	Corrosion resistance Good chemical resistance Good wear resistance	Short pot life Low maintainability depending on quality
Polyurethane	Good weather resistance High variability Fast drying response	Short pot life at high temperature Whitening when affected by moisture during curing
Fluororesin	Good weather resistance Quick drying	Short pot life at high temperature Whitening when affected by moisture during curing
Polyester resin	High-wear resistance	Short pot life
Tar epoxy resin	Water resistance Seawater resistance High-chemical resistance	Low-weather resistance Prone to whitening
Cement-based protection material	Good weather resistance Good durability	Prone to whitening

**Table 2:** Types of structure protection materials.

Components	Types of Structure Restoration Materials (Repair Mortar)
Polymer cement mortar	Polymer cement mortars such as SBR-, EVE-, and PAE-based (including rust inhibitor addition-based). The use of pre-combined products containing re-emulsifying powder resin is increasing
Polymer mortar	Polymer mortars (resin mortars), such as polyester resin-based, epoxy resin-based, acrylic resin-based (there are many products using lightweight aggregates)
Cement mortar or concrete	Ordinary cement mortar or concrete mixed with cement, such as ordinary Portland cement, high-early strength Portland cement, and rapidly set cement, aggregates, and chemical admixture for concrete

**Table 3:** Types of crack-repair materials (crack-injection materials).

Components	Types of Crack-Repair Materials (Injection Materials)
Resin-based	Epoxy resin for injection, flexible epoxy resin for injection, polymer mortar, etc.
Cement-based	Polymer cement paste (slurry), expanded cement injection material, etc.
Sealant	Silicon-based, urethane-based, polysulphide-based, etc.

**Table 4:** Test standards and methods for structure protection material [15,16,17,18,19,20].

Classification	Test Type	Test Standards	Test Method
Structure protection material	Accelerated carbonation	KS F 2584 [15]	Standard test method for accelerated carbonation of concrete perform tests using concrete test standards by fabricating ∅100 × 200 specimens
		KS F 2596 [16]	Method for measuring the carbonation depth of concrete
	Resistance to chloride ion penetration	KS F 2711 [17]	Standard test method for resistance of concrete to chloride ion penetration based on electrical conductance Perform tests using concrete test standards by fabricating ∅100 × 50 specimens
		American Society for Testing and Materials (ASTM) C 1202 [18]	Standard test method for electrical indication of concrete’s ability to resist chloride ion penetration Perform tests using concrete test standards by fabricating ∅100 × 50 specimens
	Resistance to freezing and thawing	KS F 2456 [19]	Standard test method for resistance of concrete to rapid freezing and thawing Perform tests using concrete test standards by fabricating ∅100 × 200 specimens
		ASTM C 666 [20]	Standard test method for resistance of concrete to rapid freezing and thawing Perform tests using concrete test standards by fabricating ∅100 × 200 specimens

**Table 5:** Test standards and methods for structure restoration materials [17,18,21,22,23,24,25,26].

Classification	Test Type	Test Standards	Test Method
Structure restoration material	Flexural strength	KS F 4042 [21]	Polymer modified cement mortar for maintenance in concrete structure—flexural strength test
		ASTM C 348 [22]	Standard test method for flexural strength of hydraulic–cement mortars
	Compressive strength	KS F 4042 [21]	Polymer modified cement mortar for maintenance in concrete structure—flexural strength test
		ASTM C 39 [23]	Standard test method for compressive strength of cylindrical concrete specimens
	Bond strength	KS F 4042 [21]	Polymer modified cement mortar for maintenance in concrete structure—bond strength test
		ASTM C 1404 [24]	Standard test method used for bond strength of adhesive systems with concrete measured by direct tension
	Resistance to chloride ion penetration	KS F 2711 [17]	Standard test method for resistance of concrete to chloride ion penetration based on electrical conductance
		ASTM C 1202 [18]	Standard test method for electrical indication of concrete’s ability to resist chloride ion penetration
	Splitting tensile strength	KS F 2423 [25]	Standard test method for splitting tensile strength of concrete
		ASTM C 496 [26]	Standard test method for splitting tensile strength of cylindrical concrete specimens

**Table 6:** Test standards and methods for crack repair material [27,28,29,30,31,32,33,34].

Classification	Test Type	Test Standards	Test Method
Crack injection material	Bond strength	KS F 4923 [27]	Epoxy adhesives for repairing in concrete structure—bond strength
		ASTM C 882 [28]	Standard test method for bond strength of epoxy-resin systems used with concrete by slant shear
	Tensile strength (elongation)	KS F 4923 [27] KS M ISO 527 [29]	Epoxy adhesives for repairing in concrete structure; tensile strength and elongation at tensile failure Plastics—determination of tensile properties—general principles (part 1), test conditions for molding and extrusion plastics
		KS M 3015 [30]	Testing methods for thermosetting plastics
		ASTM D 638 [31]	Standard test method for tensile properties of plastics
	Compressive strength	KS F 4923 [27]	Epoxy adhesives for repairing in concrete structure—compressive strength of rigid epoxy resin
		KS M ISO 844 [32]	Rigid cellular plastics—determination of compression properties
		KS M 3015 [30]	Testing methods for thermosetting plastics
		ASTM D 695 [33]	Standard test method for compressive properties of rigid plastics
	Flexural strength	KS M 3015 [30]	Testing methods for thermosetting plastics
		ASTM D 790 [34]	Standard test methods for flexural properties of unreinforced and reinforced plastics and electrical insulating materials

## Data Availability

The data presented in this study are available on request from the corresponding author.
